# Neurogenic Differentiation of Human Dental Pulp Stem Cells on Graphene-Polycaprolactone Hybrid Nanofibers

**DOI:** 10.3390/nano8070554

**Published:** 2018-07-21

**Authors:** Hoon Seonwoo, Kyung-Je Jang, Dohyeon Lee, Sunho Park, Myungchul Lee, Sangbae Park, Ki-Taek Lim, Jangho Kim, Jong Hoon Chung

**Affiliations:** 1 Department of Industrial Machinery Engineering, College of Life Science and Natural Resources, Sunchon National University, Sunchon 57922, Korea; uhun906@gmail.com; 2 Department of Biosystems & Biomaterials Science and Engineering, Seoul National University, Seoul 151-742, Korea; trudwp@naver.com (K.-J.J.); josephmyungchul@gmail.com (M.L.); sb92park@snu.ac.kr (S.P.); 3 Department of Rural and Biosystems Engineering, Chonnam National University, Gwangju 61186, Korea; ehgus2307@gmail.com (D.L.); preference9330@gmail.com (S.P.); 4 Department of Biosystems Engineering, Kangwon National University, Chuncheon 24341, Korea; ktlim@kangwon.ac.kr; 5 Research Institute of Agriculture and Life Sciences, Seoul National University, Seoul 151742, Korea

**Keywords:** alignments, dental pulp stem cells, nanofiber, neurogenesis, reduced graphene oxide

## Abstract

Stem cells derived from dental tissues—dental stem cells—are favored due to their easy acquisition. Among them, dental pulp stem cells (DPSCs) extracted from the dental pulp have many advantages, such as high proliferation and a highly purified population. Although their ability for neurogenic differentiation has been highlighted and neurogenic differentiation using electrospun nanofibers (NFs) has been performed, graphene-incorporated NFs have never been applied for DPSC neurogenic differentiation. Here, reduced graphene oxide (RGO)-polycaprolactone (PCL) hybrid electrospun NFs were developed and applied for enhanced neurogenesis of DPSCs. First, RGO-PCL NFs were fabricated by electrospinning with incorporation of RGO and alignments, and their chemical and morphological characteristics were evaluated. Furthermore, in vitro NF properties, such as influence on the cellular alignments and cell viability of DPSCs, were also analyzed. The influences of NFs on DPSCs neurogenesis were also analyzed. The results confirmed that an appropriate concentration of RGO promoted better DPSC neurogenesis. Furthermore, the use of random NFs facilitated contiguous junctions of differentiated cells, whereas the use of aligned NFs facilitated an aligned junction of differentiated cells along the direction of NF alignments. Our findings showed that RGO-PCL NFs can be a useful tool for DPSC neurogenesis, which will help regeneration in neurodegenerative and neurodefective diseases.

## 1. Introduction

Many efforts have been made to secure stem cell sources [1]. Depending on the cell sources, the stem cells are classified as embryonic stem cells (ESCs), bone-marrow-derived stem cells (BMSCs), and adipose-derived stem cells (ADSCs). However, one critical limitation for acquiring these stem cells is the long and intense nature of the acquisition processes; this necessitates a second surgery for the donor site, which increases the risk of inflammation [2,3]. However, stem cells can be derived from exfoliated teeth, too. Unlike other stem cells, the stem cells from dental tissues, called dental stem cells, can be derived without any surgery, because deciduous teeth, wisdom teeth, or unhealthy teeth in any case need to be extracted from periodontal tissues [3]. Thus, everyone can easily acquire their own stem cells by cryopreservation of the dental stem cells after exfoliation and separation. Based on origin, the dental stem cells are classified as dental follicle stem cells (DFSCs), dental papilla stem cells (DPPSCs), periodontal ligament stem cells (PDLSCs), and dental pulp stem cells (DPSCs) [4,5,6]. Among all these types of dental stem cells, DPSCs extracted from the dental pulp have many advantages. Unlike other stem cells, DPSCs are highly purified [7] and are highly proliferative over 50 passages. Furthermore, the DPSCs have a vast potency: in addition to differentiating into dental tissues [8], DPSCs can also differentiate into bone [9], muscle [10], cartilage [10], and even neuronal cells [11]. Due to these advantages, DPSCs have been used in various fields, such as research on immunodeficiency disease [8] and neurological diseases [12]. DPSCs have been shown to themselves differentiate into neuronal cells and regenerate to ameliorate neurological dysfunctions [13]. Their neurogenic differentiation is largely dependent on chemical factors [14]. In addition to chemical factors, micro- to nano-structural cues and electroconductive biomaterials can also affect the efficacies of neurogenic differentiation of DPSCs. However, the neurogenic differentiation of DPSCs with biomaterials inducing micro- to nano-structural cues and electroconductivity has rarely been investigated.

Electrospinning is used to fabricate micro- to nanometer-sized fibers by electric and hydrostatic forces [15]. The electrospun nanofibrous membrane has been widely used in tissue engineering, because its structures are quite similar to the structures of the extracellular matrix (ECM) [16]. In addition to random nanofibers (RFs), aligned nanofibers (AFs) can also be acquired by changing the characteristics of collectors [17]. The ECM can have different tissue-specific organizations [18]. Therefore, the application of NFs resembling the specific features of the ECM has shown outstanding results [16]. Graphene is composed of 1‒10-layered subsheets of graphite, which are in turn composed of SP^2^-hybridized hexagonal carbon [19]. Due to its conformational and electrochemical properties, graphene has strong mechanical properties and excellent electric properties. Such mechanical and electrochemical properties are known to influence stem cell proliferation and differentiation. Graphene and its subfamily have been reported to improve the osteogenesis, neurogenesis, epithelial differentiation, and cardiomyogenic differentiation of stem cells [20,21]. Graphene oxide (GO), one of the graphene derivatives, is usually used with NFs for tissue engineering due to its hydrophilicity, low cytotoxicity, and degradability [22]. On the contrary, reduced graphene oxide (RGO), which is derived from GO by reduction of the hydroxyl or carboxyl group [23], has rarely been used in combination with NFs, especially in neurogenesis. Electroconductive materials generally promote the neurogenesis of stem cells. However, because reduction of a hydroxyl and carboxyl group results in the recovery of π–π bonds, RGO has higher electroconductivity than GO [24]. If RGO-incorporated NFs are fabricated and applied to stem cells, the structural cues induced by the NFs and the electrochemical cues induced by RGO would synergistically enhance the differentiation of stem cells, especially for neurogenesis. However, to our knowledge, the application of RGO-incorporated NFs in DPSC neurogenesis has never been studied.

**Figure 1:**
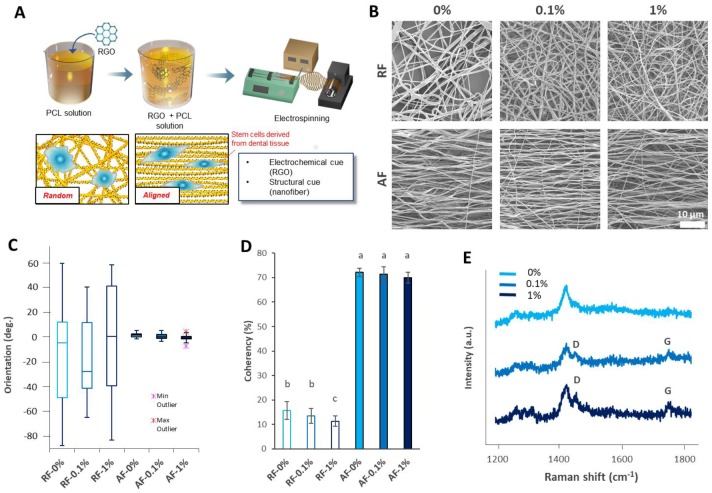
Characteristics of the reduced graphene oxide (RGO)–polycaprolactione (PCL) hybrid electrospun nanofibers (NFs) (RGO-PCL NFs). (**A**) Study strategy. RGO was incorporated into PCL solution and sonicated for even distribution. Then, the solution was electrospun to achieve random nanofibers (RFs) and aligned nanofibers (AFs). The fabricated nanofibers were used for the neurogenic differentiation of dental pulp stem cells (DPSCs). (**B**–**D**) Morphological analysis. (**B**) Representative images of electrospun RGO-PCL NFs. (**C**) Orientation of the RGO-PCL NFs. The orientation of the RFs are distributed broadly, whereas those of the AFs show narrow distribution. Each of the 25 images were used for the analysis. (**D**) Coherency of the RGO-PCL NFs. The coherencies of AFs were significantly higher than those of RFs. Each of 25 images was used for the analysis. Error bars represent the standard deviation. Same alphabets represent non-significance (*p* < 0.05). (**E**) Raman spectroscopy results. The 0.1% and 1% RGO-incorporated NFs exhibited D (~1450 cm^−1^) and G peaks (~1600 cm^−1^), the characteristic peaks of RGO. (**C**) XRD results. The 0.1% and 1%-incorporated NFs exhibited the characteristic peaks of RGO at approximately 23.5°.

Therefore, in this study, we developed RGO-polycaprolactone (PCL) hybrid NFs (RGO-PCL NFs) and applied them to the neurogenesis of DPSCs (Figure 1A). For evaluating the structural cues induced by the RGO-PCL NFs, RFs and AFs were independently fabricated. In addition, the concentration of RGO was logistically applied. Their morphology was evaluated using scanning electron microscopy (FE-SEM), and their structural properties were analyzed using Image J software. The chemical properties of the RGO-PCL NFs were analyzed by Raman spectroscopy. The influences of RGO and the NFs were observed by immunocytochemistry (ICC) and quantitatively analyzed. After neurogenic differentiation, the morphologies and alignments were also observed and analyzed. Finally, the potential of the RGO-PCL NFs for application in DPSC neurogenesis was addressed.

## 2. Materials and Methods

### 2.1. Fiber Fabrication

Electrospun NFs were obtained by electrospinning 10 wt % polycaprolactone (Mw: 80 kDa, Simga-Aldrich, St. Louis, MO, USA) solution in chloroform/*N*,*N*-dimethyl formamide (DAEJUNG, Siheung, Korea) (*v*/*v* = 3/1) incorporating 0, 0.01%, and 0.1% RGO. Random nanofibers (NFs) were deposited on planar plate and aligned nanofibers (AFs) were deposited on a custom-made rotating drum.

### 2.2. Cell Culture

The DPSCs were collected at the Intellectual Biointerface Engineering Center, Dental Research Institute, College of Dentistry, Seoul National University. All experiments related to the DPSCs were approved by the Seoul National University Institutional Animal Care and Use Committee (SNU-120427-2-2). The cells were cultured in α-minimum essential medium (MEM) containing 10% fetal bovine serum (FBS, Welgene Inc., Gyeongsan, Korea), 10 mM ascorbic acid (l-ascorbic acid, Simga-Aldrich, St. Louis, MO, USA), antibiotics (Welgene Inc., Gyeongsan, Korea), and sodium bicarbonate (DUKSAN, Seoul, Korea) at 37 °C in a humidified atmosphere of 5% CO_2_ (Steri-Cycle 370 Incubator, Thermo Fisher Scientific, Waltham, MA, USA). The medium was changed every other day. When the cells became confluent, they were detached with 1 mL trypsin-EDTA solution (LS 015-10, Welgene Inc., Gyeongsan, Korea), counted, and passaged.

### 2.3. Cell Viability Test

Cell viability was measured using a WST-1 assay (EZ-Cytox cell viability assay kit, Daeillab Service Co., Ltd., Seoul, Korea). Water-soluble formazan was quantified by a multiwell spectrophotometer (Victor 3, Perkin Elmer, Waltham, MA, USA), measured at 450 nm. For ICC, DPSCs (1 × 10^4^ cells sample-1) were seeded on the substrates and allowed to spread for 7 days in culture media at 37 °C in a humidified atmosphere containing 5% CO_2_. The adhered cells were fixed with a 4% paraformaldehyde solution (Sigma-Aldrich, St. Louis, MO, USA) for 20 min, permeabilized with 0.2% Triton X-100 (Sigma-Aldrich, St. Louis, MO, USA) for 15 min, and stained with TRITC-conjugated phalloidin (Millipore, Burlington, MA, USA) and 4,6-diamidino-2-phenylinodole (DAPI; Millipore, Burlington, MA, USA) for 1 h. Focal adhesions (FAs) were stained with a monoclonal anti-vinculin antibody (1:100; Millipore, Burlington, MA, USA) and an FITC-conjugated goat anti-mouse secondary antibody (1:500; Millipore, Burlington, MA, USA). Images were taken using a confocal laser scanning microscope (LSM710, Carl Zeiss, Oberkochen, Germany).

### 2.4. Neurogenic Differentiation

Dental pulp stem cells were placed at a density of 1 × 10^4^ cells/cm^2^ and cultured for 1 week in Mesenchymal Stem Cell Neurogenic Differentiation Medium (PromoCell, Heidelberg, Germany). ICC was conducted on day 3 and day 7 to exhibit the expression of Tuj-1 and neuronal nuclei (NeuN). The cultured cells were washed in phosphate-buffered saline (PBS, Simga-Aldrich, St. Louis, MO, USA), fixed in a 4% paraformaldehyde solution (Simga-Aldrich, St. Louis, MO, USA) for 20 min, and permeabilized with 0.2% Triton X-100 (Simga-Aldrich, St. Louis, MO, USA) for 15 min. Cells were incubated with anti Tuj-1 (abcam, Cambridge, UK), anti NeuN (abcam, Cambridge, UK), and 4,6-diamidino-2-phrnykinodole (DAPI; Millipore, Burlington, MA, USA) for 1 h.

## 3. Results and Discussions

### 3.1. Characterization of the RGO-PCL NFs

First, the RGO-PCL NFs were characterized. The morphology of RGO-PCL NFs was assessed by SEM (Figure 1B). The RFs were disordered without any alignment, whereas the AFs were aligned along with the revolution direction of the rotating collector. The alignments of the NFs were quantitatively analyzed using Image J software (National Institutes of Health, Bethesda, MD, USA). The box plot revealed that the orientations of the RFs were distributed broadly, whereas those of the AFs were concentrated in a narrow region (Figure 1C). The AFs showed significantly higher coherency than the RFs (Figure 1D). Furthermore, incorporation of RGO influenced the alignments of the NFs. One percent incorporation of RGO caused a significant decrease in the coherency of RF-1% compared with that of RF-0% and RF-0.1%. In Raman spectroscopy, the NFs with 0.1% and 1% RGO exhibited D (~1450 cm^−1^) and G peaks (~1600 cm^−1^), the representative peaks of RGO (Figure 1E).

### 3.2. Influence of the RGO-PCL NFs on DPSC Behavior

**Figure 2:**
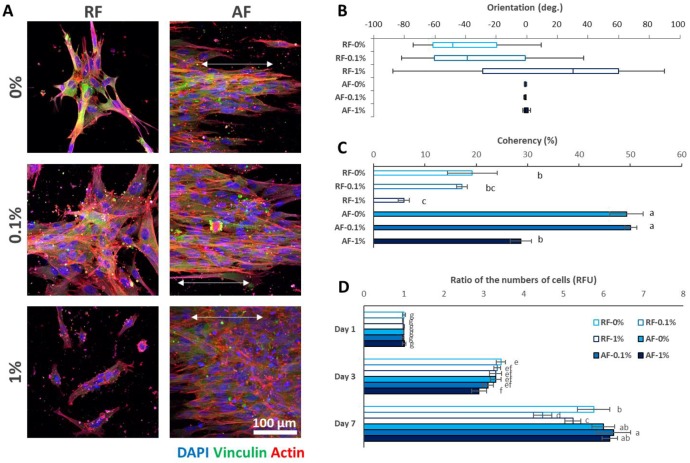
Effects of the RGO-PCL NFs on DPSC behavior. (**A**–**C**) Analyses of cellular morphologies. (**A**) Immunocytochemistry (ICC) results 2 days after seeding. Corresponding with the NF alignments, the DPSCs on the RFs were randomly aligned, whereas those on the AFs were aligned well. In particular, vinculin, an indicator of focal adhesion, did not stain the cells on the 1% RGO-PCL NFs, which indicated that excessive RGO incorporation may be harmful to initial cell adhesion. (**B**) DPSC alignments. Corresponding to the RGO-PCL NFs, the cells on the RFs showed broad orientation, whereas those on the AFs showed narrow orientation. (**C**) Coherency of the DPSC alignments. The alignments of the DPSCs on AFs were significantly higher than those of cells on the RFs, except for AF-1%. Furthermore, the cells on NFs with high RGO incorporation (1%) showed significantly decreased coherency. Error bars indicate the standard deviation. Same alphabets mean a non-significant difference between samples (*p* < 0.05). (**D**) Ratio of the numbers of DPSCs on the RGO-PCL NFs. On day 3, cells on AF-1% showed significantly decreased viability. On day 7, cells on AF-0.1% showed significantly higher viability, whereas those on RF-0.1% and RF-1% showed significantly lower viability. Error bars indicate the standard deviation. Same alphabets mean a non-significant difference between samples (*p* < 0.05).

After characterization of the NFs, their influence on DPSCs was assessed. Two days after seeding the DPSCs, the cellular morphologies were observed by ICC (Figure 2A). The DPSCs on the RFs were aligned randomly, whereas those on the AFs were well-aligned following the orientation of the fibers. The alignments of the DPSCs according to the NFs were analyzed using image analysis [25]. Corresponding to the alignments of NFs, the cellular alignments were randomly distributed on the RFs, whereas those on the AFs were narrow (Figure 2B). The AFs exhibited significantly higher coherency than the RFs, except for AF-1% (Figure 2C). Based on the results, the cellular alignments were confirmed to be influenced highly by NF alignments. Furthermore, high incorporation of RGO (1%) decreased cellular alignments significantly. In the characterization study, high incorporation of RGO (1%) decreased the alignments of the NFs. Therefore, it was anticipated that high incorporation of RGO (1%) decreased the alignments of NFs, which further resulted in a corresponding decrease in cellular alignments. The ratio of the numbers of alive cells was also assessed (Figure 2D), and it was found that the NF alignments and RGO concentration did not significantly affect the numbers of alive cells on day 3, except for AF-1%; the ratio of the numbers of cells with AF-1% was significantly decreased. Based on the results, we believe that the NF alignments and high concentration of RGO (1%) negatively affect initial cell proliferation. On the contrary, the NF alignments and RGO concentration appeared to affect the ratio of the numbers of cells on day 7. Cell numbers with AF-0.1% were significantly increased, whereas those with RF-0.1% and RF-1% were significantly decreased. It is well-known that the incorporation of nanomaterials usually has negative effects on cell viability. Concurrently, on day 7, we found that cell viability was decreased on RF-0.1% and RF-1%. On the contrary, the cell viability on AF-1% was similar to that on RF-0%. The cell viability on AF-0.1% was further significantly higher than that on RF-0.1%. Thus, we believe that incorporation of an appropriate percentage of RGO and AFs synergistically increases the cell numbers of DPSCs.

### 3.3. Neurogenic Differentiation

**Figure 3:**
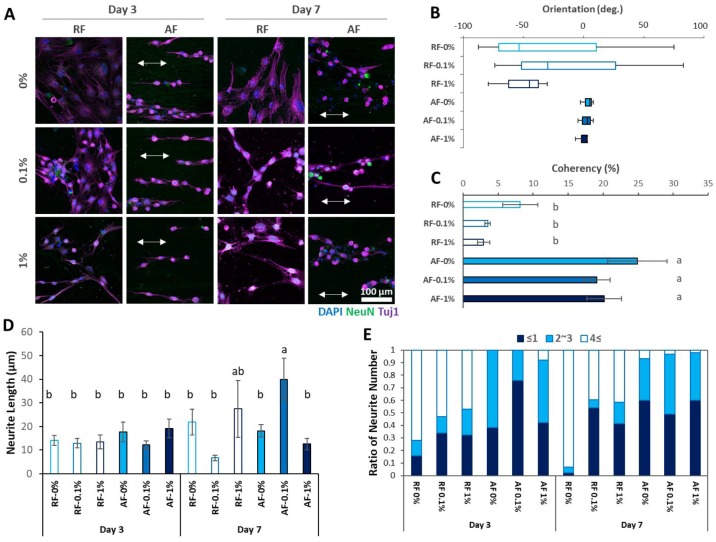
Neurogenic differentiation of DPSCs using RGO-PCL NFs. (**A**) ICC results. The RGO-incorporated NFs resulted in faster morphological transformation and early expression of Tuj-1 and NeuN. The DPSCs on the RFs seemed randomly aligned, whereas those on the AFs seemed well-aligned. (**B**) Orientation of the alignments of the differentiated cells. Corresponding to the alignments of the RGO-PCL NFs, the cells on the RFs showed broad orientation, whereas those on the AFs showed narrow orientation. (**C**) Coherency of the alignments of the differentiated cells. The alignments of DPSCs on the AFs were significantly higher than those of the DPSCs on the RFs, except for AF-1%. Furthermore, the cells with high RGO incorporation (1%) showed significantly decreased coherency. Error bars indicate the standard deviation. Same alphabets mean a non-significant difference between samples (*p* < 0.05). (**D**) Neurite length of the differentiated cells. The AF-0.1% group showed significantly increased neurite length compared with other groups. Error bars indicate the standard deviation. Same alphabets mean a non-significant difference between samples (*p* < 0.05). (**E**) Distribution of neurite numbers on each group. RF groups and AF groups showed apparent changes in distribution of neurite numbers.

The effects of RGO and NFs on the neurogenic differentiation were also observed. The DPSCs seeded on the RGO-PCL NFs were subjected to neurogenic differentiation. On the 3rd and 7th day, the DPSCs seeded on NFs with 0.1% and 1% RGO showed apparent changes in their morphologies (Figure 3A). In contrast to the cells on tissue culture polystyrene (TCPS) (Figure S1), those grown on RGO-PCL NFs severely changed their morphology, representing that the RGO-PCL NFs promote neurogenic differentiation of DPSCs compared to the TCPS. In particular, NFs with 0.1% and 1% RGO resulted in high expression of Tuj-1, the early marker of neurogenesis, and NeuN, the late marker of neurogenesis. In particular, the expression of NeuN was visually compared in Figure S2. As a result, the intensity of NeuN expression seemed to be increased on higher RGO concentration groups or AF groups. Therefore, the neurogenic differentiation of the DPSCs can be influenced by the concentration of nanomaterials and alignment of adjacent ECM. In addition, the cells on the 1% group showed shorter axon-like legs. These results indicated that excessive incorporation of RGO (1%) might result in neurodegeneration of DPSCs. Therefore, incorporation of the appropriate concentration of RGO (0.1%) might promote the neurogenic differentiation of DPSCs. The alignments of the NFs also affected the orientation of the differentiated cells. The alignments of cells on the RFs were randomly distributed, whereas those on the AFs were highly organized. Based on the ICC results, we conducted image analyses to confirm the cellular alignments (Figure 3B,C). The differentiated cells on the RFs exhibited randomly oriented alignments, whereas those on the AFs exhibited well-oriented alignments (Figure 3B). The coherency also proved the result: those on the AFs showed significantly better results than those on the RFs (Figure 3C). The neurite length of each group was assessed (Figure 3D). The AF-0.1% group on day 7 showed significantly increased neurite length compared with other groups, except for RF-1% on day 7. Consequently, appropriate RGO incorporation and aligned ECM may increase neurite extension.

**Figure 4:**
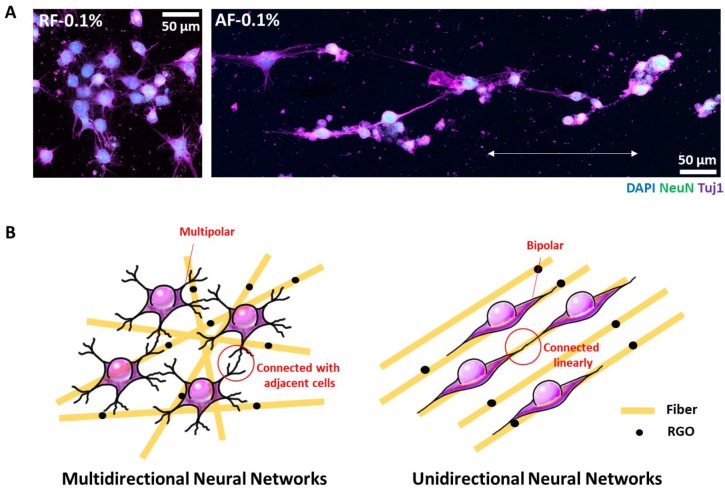
Effects of the RGO-PCL NF alignments on the conjunction of the differentiated DPSCs. (**A**) Comparison of neurites. Neurites differentiated from cells on RF-0.1% were connected with the adjacent cells, whereas those on AF-0.1% stretched and connected along the direction of the AF alignments. (**B**) Perspectives of the study. Because RF-0.1% can connect the differentiated cells with the adjacent cells, it can be used in the generation of multidirectional neural networks. On the other hand, AF-0.1% can be used in the generation of unidirectional neural networks, because it can align and connect the differentiated cells along the direction of the AF alignments.

The alignments of the NFs seemed to influence not only the cellular alignments, but also cellular morphologies. The cells on RF-0.1% had multipolar structures, whereas those on AF-0.1% had bipolar structures (Figure 4A). Therefore, it was proposed that the cells on RF-0.1% were connected with the neighbor cells, whereas those on AF-0.1% seemed to be connected along the direction of the fiber alignments. When we compare the ratio of neurite numbers, cells grown on RFs showed apparently higher neurite numbers compared to those grown on AFs (Figure 3E). It is well-known that nanostructures affect cellular adhesion and alignments [26]. In particular, anisotropic nanopatterns give rise to well-ordered alignments with adjacent cells [27,28]. Cellular alignments influence not only cellular function, but also stem cell differentiation. Therefore, anisotropic nanopatterns have been used frequently in stem cell engineering, especially in neurogenic differentiation. To date, many types of techniques, such as self-assembly [29], lithography [30], and electrospun NFs [31], have been used in neurogenesis. Among them, electrospun NFs have been used widely due to their good biocompatibility and easy fabrication. Furthermore, specific cytokines or nanomaterials can be easily incorporated into the NFs, which results in enhanced functionality of the NFs. To date, many nanomaterials have been reported to affect cellular behaviors [32,33,34]. Among them, GO- and RGO-incorporated NFs have been reported to enhance the viability of neuronal cells and mesenchymal stem cells, respectively. However, the influence of RGO-incorporated NFs on stem cell differentiation, especially in neurogenic differentiation, has not been studied so far. The results of our study showed that the incorporation of an appropriate concentration of RGO (0.1%) increases cell viability and neurogenic differentiation. Furthermore, the alignments of the NFs influence the alignments of the DPSCs as well as the linkage of differentiated neurites. Based on these results, we suggest that the use of RF-0.1% is suitable for the generation of multidirectional neural networks, whereas the use of AF-0.1% is suitable for the generation of unidirectional neural networks (Figure 4B).

## 4. Conclusions

In this study, RGO-PCL NFs were fabricated with different alignments and RGO concentrations and applied to the neurogenesis of DPSCs. The presence of RGO was confirmed by Raman spectroscopy and XRD. The alignments of the RGO-PCL NFs directly affected the alignments of the DPSCs: the DPSCs followed the alignments of the RGO-PCL NFs. Furthermore, the combination of the alignments and RGO increased the cell viability. In the neurogenic differentiation study, incorporation of an appropriate concentration of RGO (0.1%) enhanced the neurogenesis of the DPSCs. Furthermore, the alignments of NFs seemed to correlatively affect the tissue morphologies. In conclusion, our findings indicated that the application of RGO-PCL NFs with an appropriate concentration of RGO would open the gates for the use of DPSCs in neurological therapy and neurogenesis.

## Supplementary Materials

The following are available online at http://www.mdpi.com/2079-4991/8/7/554/s1. Figure S1. Neurogenic differentiation of DPSCs on TCPS. Compared to the NF groups, the TCPS groups showed less differentiated cell shapes and the expression of neurogenic markers, Figure S2. NeuN expression of each condition. The more the RGO concentration, the higher the NeuN expression. Furthermore, the expression of NeuN was higher on the AF groups compared to the RF groups.
