# Supplementary material for: Neurogenic Differentiation of Human Dental Pulp Stem Cells on Graphene-Polycaprolactone Hybrid Nanofibers
Source: Nanomaterials (Basel). 2018 Jul 21;8(7):554. doi: 10.3390/nano8070554 (PMC6071115; doi:10.3390/nano8070554)
Supplement: Supplementary file 1 [file nanomaterials-08-00554-s001.pdf]

Neurogenic Differentiation of Human Dental Pulp Stem Cells on Graphene-Polycaprolactone Hybrid Nanofibers

Hoon Seonwoo [1,†], Kyung-Je Jang [2,†], Dohyeon Lee [3], Sunho Park [3], Myungchul Lee [2], Sangbae Park [2], Ki-Taek Lim [4], Jangho Kim [3,*], and Jong Hoon Chung [2,5,*]

Supporting Information

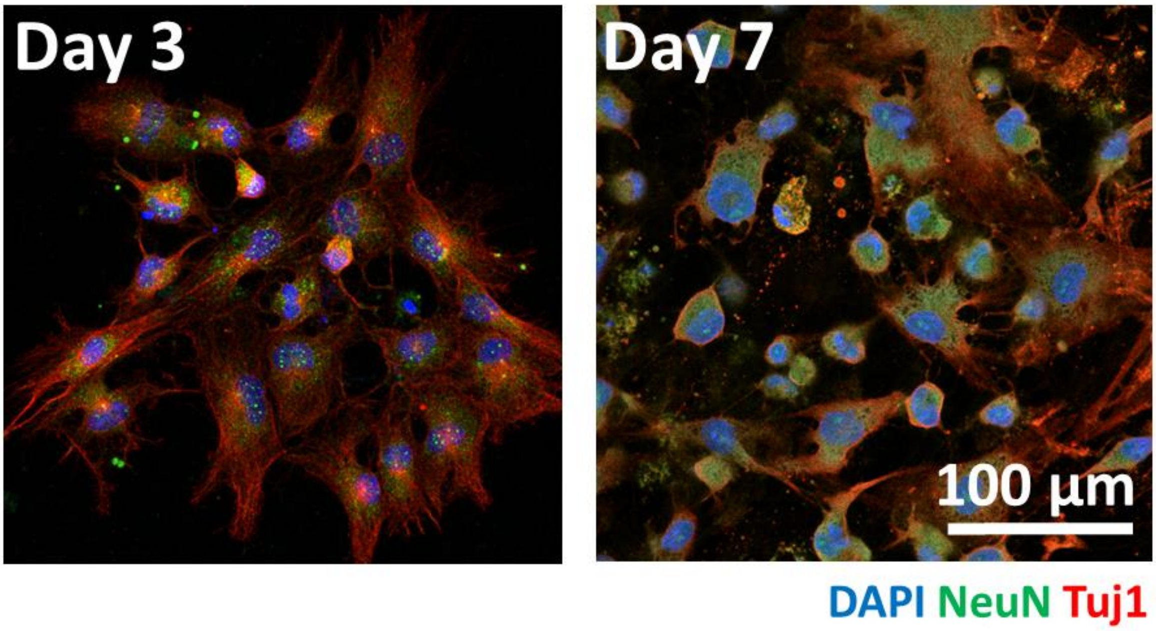

Figure S1. Neurogenic differentiation of DPSCs on TCPS. Compared to the NF groups, the TCPS groups showed less differentiated cell shapes and the expression of neurogenic markers.

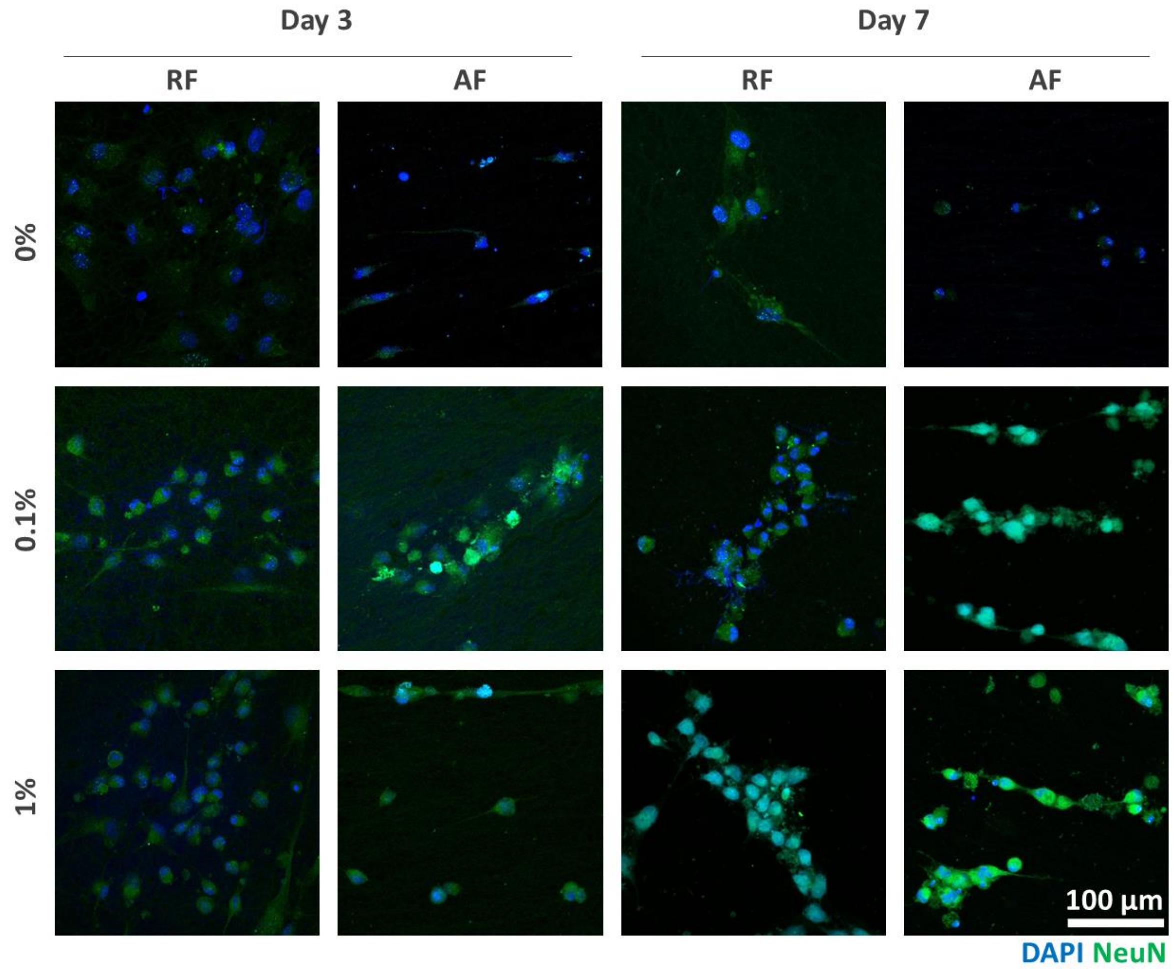

Figure S2. NeuN expression of each condition. The more the RGO concentration, the higher the NeuN expression. Furthermore, the expression of NeuN was higher on the AF groups compared to the RF groups.
